# Endocannabinoid Signaling in Midbrain Dopamine Neurons: More than Physiology?

**DOI:** 10.2174/157015907782793612

**Published:** 2007-12

**Authors:** M Melis, P Pistis

**Affiliations:** B.B. Brodie Department of Neuroscience and Center of Excellence for the Neurobiology of Addiction, University of Cagliari, Monserrato (CA), 09042, Italy

**Keywords:** Dopamine, endocannabinoid, synaptic plasticity, neuroprotection, reward, schizophrenia, Parkinson,s disease.

## Abstract

Different classes of neurons in the CNS utilize endogenous cannabinoids as retrograde messengers to shape afferent activity in a short- and long-lasting fashion. Transient suppression of excitation and inhibition as well as long-term depression or potentiation in many brain regions require endocannabinoids to be released by the postsynaptic neurons and activate presynaptic CB1 receptors. Memory consolidation and/or extinction and habit forming have been suggested as the potential behavioral consequences of endocannabinoid-mediated synaptic modulation.

However, endocannabinoids have a dual role: beyond a physiological modulation of synaptic functions, they have been demonstrated to participate in the mechanisms of neuronal protection under circumstances involving excessive excitatory drive, glutamate excitotoxicity, hypoxia-ischemia, which are key features of several neurodegenerative disorders.

In this framework, the recent discovery that the endocannabinoid 2-arachidonoyl-glycerol is released by midbrain dopaminergic neurons, under both physiological synaptic activity to modulate afferent inputs and pathological conditions such as ischemia, is particularly interesting for the possible implication of these molecules in brain functions and dysfunctions.

Since dopamine dysfunctions underlie diverse neuropsychiatric disorders including schizophrenia, psychoses, and drug addiction, the importance of better understanding the correlation between an unbalanced endocannabinoid signal and the dopamine system is even greater. Additionally, we will review the evidence of the involvement of the endocannabinoid system in the pathogenesis of Parkinson’s disease, where neuroprotective actions of cannabinoid-acting compounds may prove beneficial.

The modulation of the endocannabinoid system by pharmacological agents is a valuable target in protection of dopamine neurons against functional abnormalities as well as against their neurodegeneration.

## INTRODUCTION

In the last 15 years, the discovery and molecular dissection of the endocannabinoid (eCB) system has opened a new avenue in neuropsychopharmacological research. The interest in this system was further fuelled by the characterization of the dual nature of its actions within the central nervous system: modulation of synaptic functions and neuroprotection. In fact, endogenous cannabinoids (endocannabinoids) were discovered to play a role in the regulation of behavioural functions, such as reward and addiction, anxiety, feeding, and in the pathophysiological mechanisms of several neurodegenerative diseases. In this review, we will focus on the interaction between endocannabinoids (eCBs) and midbrain dopamine (DA) neurons, as recent studies highlight that it spans from the fine regulation of synaptic inputs to neuroprotection/neurorescue mecha-nisms, which may bear relevance in several neuropsychiatric disorders involving primarily dysfunctions of the DA neurons.

## THE ENDOCANNABINOID SYSTEM

The eCBs are a family of lipid molecules that form a novel class of intercellular messengers, whose functions include retrograde signaling in the brain by modulating and/or mediating several types of synaptic plasticity. These molecules make up their own system (i.e. the endocannabinoid system) comprising synthesizing and inactivating enzymes, a transport protein, and the cannabinoid (CB) receptors [94, 130]. 

Among diverse endogenous lipid molecules with eCB-like activity, the best characterized eCBs – in terms of biosynthesis, cellular transport, metabolism, and biologicalfunctions- are anandamide (AEA) [44] and 2-arachidonoyl glycerol (2-AG) [133, 195], whose content is greatly elevated in response to a variety of physiological and/or pathological stimuli. Among the other eCBs, 2-arachidonyl-glyceryl ether (2-AGE, nolandin), *O*-arachidonoyl-ethanolamine (virodhamine), and *N*-arachidonoyl-dopamine (NADA) are the most investigated, although their physiological role is still unknown.

The increased levels of eCBs are usually part of an *on demand* response. In particular, the eCBs 2-AG and AEA have been shown to be synthesized *on demand* [18, 46] by the postsynaptic cell in response to either physiological and/or pathological stimuli in several brain regions. Once released, they activate CB type 1 (CB1) receptors located presynaptically, and inhibit neurotransmitter release. As a result of their highly selective reduction of synaptic inputs onto the releasing neuron(s), eCBs influence both short- and long-term forms of synaptic plasticity.

Once activated CB1 receptors, eCBs are rapidly cleared away from their extracellular targets by a specific uptake system [9, 87], named AEA membrane transporter (AMT), which is widely distri-buted throughout the brain [71]. Then AEA and 2-AG are degraded by two well-characterized enzymes, the fatty acid amide hydrolase (FAAH) and the monoacylglycerol (MAG) lipase, respectively [36, 49, 196, 201]. 

These peculiar features (i.e. *on demand* synthesis and rapid degradation) indicate that eCBs operate close to where they are synthesized, and make them as key molecules in brain functions and dysfunctions. 

New pharmacological tools haveenabled the study of the physiological roles played by eCBs, opening up new strategies in the treatment of pain,obesity, and neurological diseases like multiple sclerosis,emotional disturbances such as anxiety and other psychiatricdisorders including drug addiction. More recently, pharmaceutical research aims to develop drugs exploiting the different biological mechanisms involved in the metabolic pathways of the two best characterized eCBs, AEA and 2-AG, to treat diverse disorders [153]. 

In fact, AEA derives from the cleavage of a *N*-arachidonoyl-phosphatidylethanolamine (NAPE), a precursorsynthesized by the enzyme *N*-acyltransferase (NAT), whichrequiresthe presence of Ca^2+^ and is regulated by cAMP [18, 156]. Its release is catalyzedby a recently cloned specific phospholipase D (PLD) [83, 147], whose activity is regulated by depolarizationand/or activation of iono-tropic (e.g. NMDA, nicotinic α7 neuronal receptors; [156, 193] or metabotropicreceptors [73, 96, 208].

On the other hand, 2-AG derives from the metabolism of triacylglycerols, through receptor-dependent activation of phosphatidylinositol-specific phospholipase A_1_ (PLA_1_) and/or C (PLC) [196]. The current model, proposing that activation of metabotropic receptors coupled to the PLC and diacylglycerol (DAG) lipase pathway leads to 2-AG production [156, 194], is substantiated by both the cloning of the enzyme 1,2-DAG lipase [11] and the contribution of ionotropic purinergic receptors (e.g. P2XT; [217]) to 2-AG formation. 

Irrespective of their different *routes*, binding properties and intrinsic activity at CB receptors [86, 88, 194], both AEA and 2-AG do activate CB receptors. The CB receptors are part of the superfamily of G protein-coupled receptors.The CB1 receptor is the most abundant G protein-coupled receptor expressed in the brain [84, 90], but it is also found in diverse peripheral tissues (e.g. muscle, the gastrointestinal tract, liver, pancreas and adipose tissue) [7, 43, 84, 90, 213]. The CB2 receptor is instead found on several immune cells (e.g. monocytes, microglial cells, T- and B-cells), in the spleen and tonsils [59, 144, 156], and peripheral tissues [116]. In addition, increasing pharmacological evidence suggest the presence of CB2 receptors in the brain [5, 148, 206], as well as the existence of at least two non-CB1, non-CB2 receptors [82, 89, 103]. Lastly, pharmacological studies revealedthe existence of eCB targets other than CB receptors, including thetransient receptor potential vanilloid-1 (TRPV1) receptor [223].

Both CB1 and CB2 receptors are coupled to similar transduction systems. CB receptor activation was initially reported to inhibit cAMP formation through its coupling to G_i _proteins [43, 91], resulting in a decrease of the protein kinase A-dependent phosphorylation processes as well. However, additional studies found that the CB receptors are also coupled to ion channels through the G_olf_ protein, resulting in the inhibition of Ca^2+^ influx through N- [117], P/Q- [200] and L- [65] type Ca^2+^channels, as well as the activation of inwardly rectifying potassium conductance and A currents [32, 118]. Additionally, CB1 and CB2 receptors have been shown to be coupled to other intracellular cascades, including the mitogen-activated protein kinase cascade, the phosphatidylinositol 3-kinase, the focal adhesion kinase, ceramide signaling and nitric oxide production [14, 42, 62, 88, 142, 171]. 

The widespread presence of the eCBsystem correlates with its role as a relevant modulator of multiple physiological functions and not only in the CNS [45].A comprehensive analysis of all the functions of the eCBs is beyond the scope of the present review. The reader will find an extensive list of recent reviews that explore the physiological relevance of the eCB system elsewhere [99, 153, 165], whereas we focus on the cellular and system physiological events mediated by eCBs that are relevant to our understanding of their interplay with the DA system. 

As we better describe below, in the midbrain 2-AG is the main eCB released *on demand* after cellular depolarization and/or receptor stimulation in a Ca^2+^-dependent manner. Once produced, it acts on CB1 receptors located on both presynaptic GABAergic and glutamatergic terminals. More generally, eCBs act similarly throughout the brain [156, 177], with the end result of presynaptic inhibition of neurotransmitter release. 

This phenomenon translates in different forms of short- and long-term synaptic plasticity, depending on the involvement of GABA or glutamate transmission, respectively. 

eCBs, released upon depolarization and/or receptor activation, can transiently affect synaptic efficacy by suppressing either GABA or glutamate release, thus provoking depolarization-induced suppression of inhibition (DSI) or excitation (DSE), respectively [1, 31, 48, 216]. eCBs can also affect other forms of short term synaptic transmission, which are induced by more physiologically relevant patterns of synaptic activity [15, 17, 136], and result in modulation of synaptic strength and/or firing pattern *in vivo* [10, 29, 136].

Additional forms of eCB modulation of synaptic transmission involve the induction of long-term synaptic plasticity, namely long-term potentiation (LTP) and depression (LTD). eCBs are strongly involved in the induction of LTD, whereas their role in LTP is probably indirect *via* heterosynaptic influences, as in the hippocampus [30], and perhaps in the prefrontal cortex [106]. Both these forms of synaptic plasticity involve changes in the strength of excitatory synapses that can last from minutes to several days [112]. Because changes in synaptic strength underlie changes in postsy-naptic receptor density, and ultimately in synapse remodeling, LTP and LTD are believed to play a critical role in memory consoli-dation and behavioural learning. Consequently, eCBs participate in the adjustment of synaptic strength [31, 102, 175]. Because activation of the eCB system affects not only synaptic remodelling [42, 156], but also neuronal differentiation [172] and survival [128, 152], eCBs guarantee not only a fine regulation of information processing,but also local protective mechanisms directed at preserving brain physiological function [6, 27, 51, 95, 97, 128, 131, 137, 143, 185, 186, 209].

## ENDOCANNABINOIDS AND DOPAMINE NEURONS: PHYSIOLOGY AND NEUROPROTECTION

A detailed description of the mesencephalic DA system is beyond the scope of this review. Here it suffices to say that DA neurons in the ventral tegmental area (VTA) are involved in the pathophysiology of psychiatric disorders and drug abuse. Their axons project to forebrain areas such as the nucleus accumbens (NAc) and the prefrontal cortex. A second major subdivision of mesencephalic DA neurons are those in the more lateral substantia nigra pars compacta (SNc), which project mainly to the striatum and are deeply interconnected to other nuclei in the basal ganglia circuits, being involved in the regulation of motor functions and in the pathogenesis of Parkinson’s disease (PD).

The eCB system is emerging as an important modulator of the DA neurons. Neuronal activity of mesencephalic DA neurons is sensitive to exogenous cannabinoid agonists [57, 70]. Both Δ^ 9^-tetrahydrocannabinol and synthetic CB1 receptor agonists dose-dependently enhance firing rate and burst activity of DA neurons in the VTA, whereas their effect on SNc neurons is weaker. Enhanced electrical activity results in an increase in DA release in terminal regions, such as the nucleus accumbens [25, 198] and the prefrontal cortex [28, 158]. Under this aspect, cannabinoids display effects similar to those of other drugs of abuse belonging to different classes, that were shown to enhance DA transmission with diverse mechanisms. The levels of CB1 receptors or mRNA in the VTA and in the SNc are very low or undetectable [84, 132], thus a direct effect of cannabinoid agonists onto DA cells seems unlikely. However, several neuronal populations projecting to VTA or SNc DA neurons have been demonstrated to possess relatively large amounts of CB1 receptor mRNA, namely the glutamatergic neurons in the PFC and in the subthalamic nucleus, or the GABAergic neurons in the striatal complex as well as in the pars reticulata of the substantia nigra (SN) [129, 132]. Thus, it is conceivable that low levels of CB1 receptors are located on glutamatergic and GABAergic terminals impinging on DA neurons [127, 214], where they can fine-tune the release of inhibitory and excitatory neurotransmitter and regulate DA neuron firing.

Consistently, *in vitro* electrophysiological experiments from independent laboratories have provided evidence of CB1 receptor localization on glutamatergic and GABAergic axon terminals in the VTA and SNc. Perfusion of CB1 agonists depresses inhibitory and excitatory post-synaptic currents recorded from DA neurons [127, 138, 197]. This effect is apparently mediated by CB1 receptors located on presynaptic terminals, where they depress GABA and glutamate release onto DA cells. The presence of CB1 receptors strongly suggested a physiological role of eCBs in the modulation of synaptic functions.

This hypothesis was confirmed by patch-clamp experiments providing evidence that DA neurons release eCBs as retrograde messengers in a Ca^2+^-dependent manner. These messengers travel toward the presynaptic sites where modulate inputs by acting at CB1 receptors [136, 161].

Under what circumstances DA neurons release eCBs? In general, a state of electrical activation is the prerequisite for the eCB release. Specifically, it is triggered by depolarization of the DA neuron [138], stimulation of excitatory afferents [136], induction of burst firing *in vivo* [136] and *in vitro* [161]. These stimuli induce a cascade of intracellular events ultimately leading to an increased intracellular Ca^2+^ and release of eCBs. Under these circumstances, released eCBs transiently modulate presynaptically afferent activity and shape incoming inputs, thus inducing DSE or DSI [138, 161, 219]. The eCB 2-AG, and not AEA, is the most likely messenger in synaptic suppression in DA neurons, since the inhibition of its major synthesizing enzyme (i.e. the *sn*-1–DAG lipase) abolishes suppression of excitation [136].

The characterization of the eCB system carried out in the VTA have not been replicated yet on the more lateral SNc, thus a comparison between these two important DA regions is not possible yet. Thus, we do not currently know whether the mechanisms mentioned above are common to the whole midbrain DA neuronal population. In principle, any generalization can be misleading, since SNc DA neurons possess specific intrinsic and synaptic properties, different in many instances from those in the VTA population. In the SNc, studies demonstrated the presence of high levels of AEA and detectable levels of the putative endocannabinoid NADA [127]. Besides their agonist properties at CB1 receptors, both AEA and NADA can be considered as endovanilloids, since they also activate TRPV1 receptors at physiological concentrations, whereas 2-AG activates only CB receptors. No information is yet available on 2-AG levels specifically in the SN, which would be of interest especially when compared to those found in the VTA. In slices containing the whole mesencephalon, 2-AG concentration is ten times higher than that of AEA [137]. Although an unknown proportion of the 2-AG detected in brain tissues could be of metabolic origin, it is likely that this eCB plays a major role in synaptic modulation of SNc DA neurons. Activation of CB1 or TRPV1 receptors in the SN exerts opposing actions on synaptic afferent to DA neurons, CB1 receptors inhibit glutamate and GABA release, whereas TRPV1 receptors potentiate excitatory transmission, possibly by depolarization of axon terminals and Ca^2+^ entry [127]. Thus, a complex picture is emerging, where eCBs and endovanilloids finely tune DA neuron activity in the SN, but their contribution to the mechanisms of short-term synaptic plasticity has not yet been investigated. It is hoped that future studies will address this issue, since this is a significant gap in the research on the interaction between the dopaminergic and endocannabinoid systems, for its relevance in the control of motor activity, and in the neuropathology of PD (see below) and other movement disorders.

## FUNCTIONAL CONSEQUENCES OF ENDOCANNABINOID SIGNALING IN DA NEURONS

DA neurons fire *in vivo* in two main different patterns of activity: regular pace-maker-like activity and burst firing [75], the latter is associated to transient increases in DA release in the nucleus accumbens [74]. By using techniques with good temporal resolution (e.g. fast-scan cyclic voltammetry) it was demonstrated that burst firing is more efficacious to evoke DA release as compared to regular firing, when the average frequency was the same [74]. Thus, functional efficacy of DA neurons depends more on firing pattern (bursting *vs*. regular) then on the simple average firing frequency.

Firing rate and pattern of DA neurons depend on the activity of excitatory and inhibitory inputs (see [125] for a review and references therein), thus a feedback control of these inputs is crucial for normal functioning of DA neurons. An increase in glutamatergic transmission enhances DA neuronal activity and produces bursting pattern, which can be reproduced by local injection of glutamate [139, 149, 150]. GABAergic input is mostly from the striatal complex; it includes inputs from the NAc, the caudate nucleus, globus pallidus and ventral pallidum [77, 189, 190, 215]. An important GABAergic input arises also from interneurons in the midbrain [8, 81]. An increase in GABAergic drive reduces firing rate and decrease bursting, *via* both GABA_A_ and GABA_B_ receptors. Switching from regular to burst firing is triggered by behavioural stimuli: reward prediction error [179-182] or the identification of contextual and behavioural stimuli responsible for unpredicted events [160]. Thus, the finding that eCBs may be released during burst of DA neurons, similar to those caused by behaviourally salient events, appears particularly intriguing. These molecules may have a crucial role in setting and/or modulating signal-to-noise ratio of DA neuron activity, especially during emotional processing and sensory perception [107]. Indeed, cannabinoids strongly influence emotional processing and sensory perceptions and have been shown to perturb the emotional significance of sensory information [76, 212]. 

Disturbances of the eCB system, such as alterations in brain eCB levels or expression of CB1 receptors, induced by chronic cannabinoid intake [210] or of idiopathic origin, may be a pathophysiological substrate of neuropsychiatric disorder such as schizophrenia, and maintain or worsen their course [72, 110, 178]. Indeed, *Cannabis* use is frequent among psychotic patients [101], and there is little doubt that it may precipitate psychotic episodes and worsen the course of the disease [2, 111], possibly due to its DA-releasing properties [211]. Additional evidence suggesting a link between cannabinoids and schizophrenia is that adolescent *Cannabis* consumption was associated with an higher incidence of schizophrenia in adulthood [4, 22, 220], when corrected for confounding variables. In fact, the DA system undergoes extensive maturation and rearrangement until early adulthood: for example, DA innervation of terminal areas, such as the prefrontal cortex is not completed until late adolescence in the rat [168]; a reduced basal levels of DA and a reduced pool of readily releasable DA have been reported in adolescent rats [192]; D_1_ and D_2_ receptor binding in the striatum undergoes robust changes during adolescence as a consequence of extensive pruning of DA synapses [199]. For these reasons, the DA system may be particularly vulnerable to exogenous cannabinoid-induced disruption of the eCB system [159].

Thus, a better understanding of this neuromodulatory system is crucial in the development of pharmacological tools as a potential therapy in psychotic disorders.

## ENDOCANNABINOIDS AND NEUROPROTECTION OF DA NEURONS

Membrane depolarization or stimulation of excitatory afferents are among the better characterized mechanisms to trigger eCB release from postsynaptic neurons, including the DAergic ones. This evidence, among others, led researchers to expect that eCBs might possess neuroprotective actions [134]. In fact, either depolarization of neuronal membrane or excessively strong excitatory activity, often occurring simultaneously, can lead to or worsen neuronal damage. Neurodegeneration is the main cause of morbidity in several diseases such as Huntington’s, Parkinson’s, Alzheimer’s, motor neuron disease and stroke. Although the pathways leading to neuronal death will be different in these disorders, some similarities are likely, such as glutamate-induced excitotoxicity and damage from reactive oxygen species and toxic ion imbalances. Neuronal damage caused by toxic or ischemic insults, such as energy or oxygen deprivation, as well as traumatic injury, is strongly dependent on the release of excitatory amino acids and on rise of intracellular Ca^2+^. By depressing the strength of excitation through eCBs, neurons might reduce potential excitotoxic damage. Consistently, mice lacking the CB1 receptor gene are more susceptible to injury after stroke [154] or kainic-induced epileptic seizures [128]. It has been suggested that the release of eCBs during neuronal injury might be a protective response. Accordingly, exogenous cannabinoids have been shown to exert neuroprotection in a variety of *in vitro *and *in vivo *models of neuronal injury [56]. Their effects occur *via* different mechanisms, such as prevention of excitotoxicity by CB1-mediated inhibition of glutamatergic transmission, reduction of Ca^2+^ influx, and subsequent inhibition of deleterious cascades, TNF-alpha formation, and anti-oxidant activity [56].

The role of (endo)cannabinoids as potential neuroprotective agents in neurodegenerative diseases, of either purely genetic or multifactorial origin, has been strengthened by recent studies where changes in CB1 receptors, as well as in the levels of their endogenous ligands, have been described in animal models of PD [55] and human patients [157]. A growing body of evidence suggests that dramatic alterations of CB1 signalling occur in PD, and following levodopa treatment [16, 55]. Some of these changes might reflect compensatory mechanisms involving the plasticity of the eCB system, whereas other changes may also contribute to the pathophysiology of parkinsonism motor disorders [16].

Since eCBs depress synaptic glutamate transmission [136, 138] and limit further depolarization, their release can be envisaged as a protective mechanism by which DA neurons reduce the strength of incoming excitation. These cells are exquisitely vulnerable to excitotoxicity and oxidative stress, and this vulnerability might be partially correlated with or even be explained by the specific regulation of their excitability [100].

Studies in our laboratory provided evidence that eCBs released by DA neurons exert protective actions in a model of ischemia/reperfusion [137]. Perfusion of brain slices in oxygen and glucose deprivation for 7 minutes induced a progressive depolarization of DA neuron membrane and interruption of firing activity, which can be irreversible. The prediction that this depolarization would trigger the release of eCBs was confirmed by the finding that blockade of CB1 receptors induced a considerable worsening of the outcome of experimental ischemia [137]. Thus, eCBs might represent one of the neuroprotective mechanisms reducing DA neuronal damage during episodes of energy deprivation. The current hypothesis posits that activation of CB1 receptors by eCBs might depress glutamate release in the ischemic tissue, and consequently decrease Ca^2+^ entry and the excitotoxic damage. In keeping with previous findings, 2-AG was demonstrated to be involved in the mechanisms of neuroprotection, since inhibition of its synthesis was detrimental.

Disturbances of the eCB system might be of considerable importance in the pathogenesis of neurological disorders involving the DA system. Accordingly, dysfunctional eCB signal has been reported in PD and other movement disorders [16, 151, 170, 203]. A pharmacological intervention aimed at the enhancement of this signal might prove useful as neuroprotective therapy to reduce cell suffering/death in the early stages of these diseases. Particularly, inhibitors of MGL [122, 174], or of the putative eCB membrane transporter [38], which would enhance endogenous 2-AG levels, might prove as valuable targets, and be therapeutically useful. It is also possible that exogenous cannabinoids might mimic the eCBs by acting as neuroprotective agents in neurodegenerative diseases. This hypothesis is based, among others, on the observation that cannabinoids protect neurons from toxic insults such as glutamatergic excitotoxicity [184], ischemic stroke [145], hypoxia [188], trauma [152], oxidative stress [128], ouabain-induced secondary excitotoxicity [204, 205]. Most of these protective effects appear to be mediated by the activation of the CB1 receptors [154], although the contribution of other different mechanisms (i.e., antioxidant and/or anti-inflammatory properties) cannot be ruled out [78, 79, 89]. Cannabinoids were also shown to be neuroprotective in animal models of PD, mainly for their known antioxidant and CB1 receptor-independent properties [63, 105], but an effect through activation of CB1 receptors cannot be excluded. We found that the CB1 agonist WIN55212 exerted neuroprotective actions on DA cells subjected to ischemia, but the protection was apparent at the lowest concentrations tested (1-10 nM), whereas higher concentrations (0.1-1 μ M) were detrimental [137]. These results support the idea that CB1 receptor agonists exert biphasic effects, with low doses being the most effective. This narrow concentration-response curve might explain why other studies found exogenous cannabinoids, such as HU210, neurotoxic to mesencephalic DA neurons in culture at higher concentrations (1 μM) [98]. The reason for negative effect of moderate to high concentrations of CB1 agonists is not currently known. One possibility is that high concentrations of CB1 agonist may disrupt the balance between suppression of glutamate and GABA release, by favouring the latter and therefore reducing or reversing their neuroprotective actions. The study by Kim *et al.* (2005) found also that exogenously applied AEA was detrimental to the survival of DA neurons. However, neurotoxic actions of AEA were exerted through activation of TRPV1 receptors, where it acts as a weak agonist. This finding adds a further matter of complexity in the balance between neuropretective/neurotoxic action of (endo) cannabinoids, since it is expected that activation of TRPV1 receptor by mixed eCB/endovanilloid substances (such as anandamide and NADA) may promote cell death through Ca^2+^ entry [98] and/or stimulation of glutamate release [127].

## ENDOCANNABINOID SIGNALING IN REINFORCEMENT AND ADDICTION 

The discovery that several lipid molecules with eCB-like activity are released by midbrain DA neurons (i.e. AEA, 2-AG, NADA; [126, 127, 136, 137] has highlighted their role in modulating the reward pathway, and consequently opened new avenues in both understanding and treating drug addiction (for a recent review see [124]. In fact, the eCBs released by VTA DA neurons, by moving retrogradely toward presynaptic CB1 receptors located on both glutamatergic and GABAergic terminals [136, 138, 161, 219], finely adjust the balance between excitatory and inhibitory synaptic inputs, thus contributing to the regulation of their own firing pattern and/or activity and, consequently, of the whole reward pathway. Since activation and inhibition of this circuit correlates with drug- seeking/taking behaviours and withdrawal/drug craving, respectively, the eCBs by affecting DA neuronal function might participate in these behaviours as well. In addition, the findings that eCBs release and CB1 receptor activation are necessary for LTD in both the nucleus accumbens [162, 163] and the dorsal neostriatum [67-69] support their involvement in those behaviours mediating the crucial transition from the reward-dependent form of drug-taking to the compulsive one. This hypothesis is supported by the evidence that both pharmacological and genetic impairment of CB1 receptors inhibit motivated behaviours [33-35, 39-41, 52, 53, 108, 109, 123, 146, 176] as well as acquisition of natural rewards [166] and drug-induced increased DA neurotransmission [26, 93, 155]. Additionally, the finding that CB1 receptors might contribute to human vulnerability to addiction [221] clearly indicates that the eCB system is a crucial substrate in the neurobiology of drug addiction.

Many mechanisms of action have been proposed for the interactions between blockade of eCBs’ action and treatment of drug addiction [64, 107, 114, 124], and they all over-emphasize the interplay between the DA and eCB and/or opioid systems. However, while the DA system appears to be involved more than others in the pathophysiology of addiction, the studies examining eCB actions in the reward pathway have not fully elucidated the mechanisms underlying their involvement in the several aspects of drug dependence (e.g.: eCBs released in the VTA and primary rewarding effects of diverse drugs of abuse; eCB role in relapse to drug-seeking behaviours). The most difficult part is, in fact, to interpret the observations regarding eCB actions in the VTA [136, 138, 161]. How can we explain their role in the rewarding properties of drugs of abuse, when they can simultaneously inhibit both GABAergic and glutamatergic synaptic inputs onto VTA DA neurons? One possibility is that eCBs released by VTA DA neurons might selectively regulate their own synaptic inputs depending upon the relative level of synaptic activation of the different pathways. Thus, one possible scenario might be that when cortical afferents to the VTA are activated, producing long-lastingadaptations in DA cells that contribute to the development and maintenance of behavioural sensitization and, therefore, to drug addiction [164, 173, 202, 207, 218], eCBs might selectively act on and dampen these glutamatergic inputs [136]. This intriguing scenario might explain eCB key role in the common neurobiological substrate of motivation and addiction, and suggest how CB1 receptor blockade has proven useful in the treatment of drug addiction. Hence, despite of the many and complex neurobiological mechanisms thought to be at the basis of drug addiction, it is noteworthy to stress the undeniable usefulness of CB1 receptor blockade as promising efficacious treatment of addiction to the diverse substances, irrespective of the mechanism of action. It is of particular interest, in fact, that pharmacological blockade of CB1 receptors with SR141716A (i.e. rimonabant) prevents reinstatement and/or relapse to drug-seeking behaviour [3, 34, 35, 41, 52, 53, 60] whereas positive modulation of CB1 receptors enhances the effects of sub-threshold doses of the same drugs [3, 41, 61, 191]. Thus, it appears compelling, and promising as well, to develop pharmacological tools aimed at activating the eCB system in order to both prevent relapse to drug use and treat drug addiction.

## ENDOCANNABINOIDS IN MOTOR COORDINATION AND PARKINSON'S DISEASE 

The observations that synthetic cannabinoids, as well as hemp-derived ones, and eCBs have powerful inhibitory effects on motor activity [37, 58, 169], together with the discovery that both eCBs and CB1 receptors are abundantly distributed in the basal ganglia [47, 84, 85], strongly suggest that this system is involved in the regulation of motor behaviour as well as basal ganglia-related movement disorders, such as PD. 

Beside the changes observed in CB1 receptors within the basal ganglia during normal aging [119, 121, 167], changes in these receptors in the postmortem basal ganglia of humans affected by PD have also been demonstrated [104]. Some of these changes are likely to reflect compensatory mechanisms by which plasticity in the eCB signaling might be invoked in, or contribute to the pathophysiology of parkinsonism motor complications [16, 187]. Additionally, like many other neurological disorders, PD is accompanied by excitotoxicity, Ca^2+^ imbalance, and oxidative stress that lead to progressive neuronal death [50, 135]. As already mentioned above, SNc DA neurons are particularly exposed to oxidative stress because the metabolism of DA gives rise to several molecules that can act as endogenous toxins [113]. Thus, one plausible speculation is that -once released by these DA cells- eCBs might act not only as neuroprotective but also as neurorescue molecules to provide protection against the progression of neuronal injury characteristic of PD. However, it is still unknown whether a phenomenon similar to the one occurring in the VTA during energy deprivation [137] takes also place in the SNc. But if this is the case, the most likely scenario is that SNc DA cells would release eCBs to dampen the hyperactivated corticostriatal glutamatergic transmission, a feature of experimental models of PD [19, 20, 23, 183]. Hence,AEAfound in SNc slices, which inhibits presynaptic glutamate release in this area [126] and the striatum as well [66, 92], would act similarly to ionotropic glutamate receptors antagonists and improve symptoms of experimentalPD [24]. Accordingly, increased levels of AEA [47, 54, 80, 115] together with a downregulation of AMT and FAAH have been found in an experimental model of PD [54, 80, 115], indicating that the eCB system undergoes complex plastic changes leading to/during PD. Altogether, the above mentioned findings allow to suggest that targeting specifically the enzyme FAAH (i.e. pharmacological inhibition) might prove beneficial/useful as a novel approach to treat the abnormalcorticostriatal glutamatergic activity observed inPD. Of particular interest is also that enhancement of protective eCB signaling can be achieved through a dual, although selective, pharmacologicalinhibition of the transporter and FAAH respectively [95]. Karianan *et al.*, in fact, indicate how disruption of the two distinct mechanisms of eCB inactivation, combining transport and FAAH inhibitors AM404 and AM374, causesadditive effects mediated by potentiation of eCB tone acting on CB1 receptors.Accordingly, by enhancing AEA availability, through inhibition of AMT and FAAH, most deficits (e.g. akinesia and sensorimotor orientation) were ameliorated in an experimental model of PD [54]. 

Interestingly, levodopa treatment reverses the abnormalities of the eCB system in those experimental models of PD, where DA depletion is obtained by injecting either 6-OHDA or MPTP in the striatum [104, 115, 120, 168]. Therefore, one might speculate that the eCB system is under negative control of DA transmission, and its involvement is part of an attempt to counteract the increased GABAergic signaling in the globus pallidus, another component of the unbalanced basal ganglia physiology contributing to PD symptoms [12, 13, 21]. In sharp contrast, a different model of PD (i.e. reserpine-induced PD), where 2-AG levels were found increased in the globus pallidus [47], suggested that enhanced eCBs levels in this area contribute to PD symptom generation instead, and that selective CB1 receptor blockade might be therapeutically useful. However, CB1 receptor blockade in non-human primates failed to alleviate PD symptoms [140, 141]. It is worth mentioning that differences in the basal ganglia physiology between primates and rodents, and/or different models of experimental PD might account for opposing results found in literature.

Noteworthy, CB1 receptor stimulation exerts a biphasic effect not only on movement [222] but also on protection (see above). Therefore, it is important to keep in mind that a narrow “therapeutic window” might prove useful for treatment of PD symptoms, whether this is obtained by either enhancing eCB availability through inhibition of their uptake and/or hydrolysis or administering CB1 receptor agonists within the proper dose range.

## CONCLUSIONS

In summary, accumulating evidence indicate that eCBs are important modulators of DA neuron functions. Dysfunctions of DA neurons lead to several very frequent invalidating diseases, such as schizophrenia or PD, which also still represent an unmet clinical need. It is therefore crucial to understand how the eCB system is involved in the normal physiological regulation of the DA system, as well as if it plays a role in the in the pathophysiological mechanisms of DA neurons’ diseases. Cannabinoids have unfavorable side effects that strongly limit their clinical usefulness. However, in recent years several compounds which modulate the eCB system, without directly activating CB1 receptors, have been developed. Preclinical studies indicated that they may possess a more favorable pharmacological profile, but their possible evaluation in clinical studies is still very distant. In future, studies are needed to better characterize the role of the eCB system in pathology, in particular their involvement in schizophrenia, or to resolve the controversial issue of neuroprotective *vs*. neurotoxic effect of exogenous or endogenous cannabinoids.
